# Information Storage in a Black Hole’s Gravitational Field

**DOI:** 10.3390/e27080870

**Published:** 2025-08-16

**Authors:** Dongshan He, Jinfang Li, Qian Qiu

**Affiliations:** College of Physics & Electronic Engineering, Xianyang Normal University, Xianyang 712000, China

**Keywords:** information loss problem, black hole entropy, gravitational field entropy

## Abstract

The key to resolving the black hole information loss paradox lies in clarifying the origin of black hole entropy and the mechanism by which black holes store information. By applying thermodynamic principles, we demonstrate that the entropy of a gravitational field is negative and proportional to the strength of the field, indicating that gravitational fields possess information storage capacity. For Schwarzschild black holes, we further demonstrate that information conventionally attributed to the black hole’s interior is in fact encoded within its external gravitational field. During black hole evaporation, the emitted particles transmit this information via gravitational correlations. This study advances our understanding of gravitational field entropy and provides valuable insights toward resolving the black hole information loss problem.

## 1. Introduction

In the early 1970s, Bekenstein’s seminal conjecture proposed that black holes must possess entropy, lest the second law of thermodynamics be violated [1]. Hawking subsequently determined the area entropy of black holes, and Hawking radiation was first discovered [2,3]. In Hawking’s original theory, regardless of the initial state of the matter that collapses to form a black hole, the black hole will evaporate in the form of thermal radiation and ultimately evolve into a thermal state. This evolution implies that distinct initial states (pure or mixed) all evolve into an identical thermal final state [4], directly violating the unitarity principle of quantum dynamics for isolated systems and creating a conflict between quantum mechanics and general relativity. This issue is commonly referred to as the “black hole information loss paradox [5].” Over the past decades, numerous approaches have been proposed to resolve this paradox [6,7,8,9]; however, a universally accepted solution remains elusive.

The first category of viewpoints posits that information is lost. Hawking was a prominent early advocate of this view; Unruh and Wald have proposed that the evolution from a pure state to a mixed state during black hole collapse and evaporation does not conflict with established physics, and thus they argue that information loss is acceptable [10]. The second viewpoint maintains that information is conserved within the semi-classical framework [11,12,13]. Some scientists suggest that black holes may not completely evaporate and could leave behind remnants or transform into a “baby universe” [14,15] to preserve the relevant information. This idea holds a certain degree of plausibility and remains an active area of research.

Using the WKB approximation and energy conservation, Parikh and Wilczek developed a tunneling model for Hawking radiation, naturally yielding non-thermal radiation spectra [16]. Based on the non-thermal black hole radiation spectrum, it has been demonstrated that this spectrum not only contains correlations capable of carrying information but also that the total entropy during the black hole radiation process (i.e., the sum of the entropy of the remaining black hole and the radiation) is conserved [17,18,19]. When a black hole emits radiation, this information is carried away by the emitted particles.

However, the question of where information is stored before being emitted remains unresolved. Wheeler argued that information is stored inside the black hole and is not lost; however, the event horizon prevents external observers from retrieving it. According to this perspective, when a black hole has no radiation or has not yet completed its radiation, information is still stored inside the black hole. However, Zhang et al. have argued that the degrees of freedom inside a black hole may be insufficient to store all the information [20,21]. Thus, the location of information storage prior to emission remains uncertain. Given that black hole entropy scales with its horizon area, a prevailing view holds that information is stored on the event horizon; this is supported by studies of area quantization and entanglement entropy [22,23,24]. Hawking and collaborators proposed the existence of “soft hairs” on black hole horizons, which can encode information [25]. When the “soft hairs” on the event horizon of a black hole are excited, they can explain the area entropy of the black hole. However, it is still unclear whether these “soft hairs” carry information about the initial state of the black hole or whether such information can be obtained by external observers.

The definition and nature of gravitational field entropy constitute a fundamental question in cosmology, inspiring numerous attempts to derive black hole entropy [26,27,28]. Since entropy is a fundamental concept in thermodynamics, it offers insights into the microstructure and information content of physical systems. Consequently, the study of gravitational field entropy represents a key area of research in theoretical physics, particularly within cosmology and black hole physics [29]. Understanding gravitational entropy holds the potential to unravel profound mysteries in black hole physics and the nature of spacetime itself.

For an isolated ideal gas, it will eventually attain a state of uniform mixing, where the maximum entropy is achieved. However, under the influence of gravity, such as on Earth, this uniformly mixed gas system evolves into an inhomogeneous distribution described by the Boltzmann distribution, where gas density increases with gravitational strength, making the system inhomogeneous [30]. Consequently, the gas entropy decreases during this spontaneous process. To satisfy the second law of thermodynamics, entropy must increase elsewhere in the system. Critically, the gravitational field is the only component undergoing change during this process, suggesting that entropy can be stored within it. Thermodynamically, the entropy of photon gas (thermal radiation) is given by $S∼T^{3}$ *V*. Given that thermal radiation can be viewed as the superposition of an infinite ensemble of plane electromagnetic waves, the entropy associated with thermal radiation underscores the fact that the electromagnetic field possesses entropy. This analogy suggests that the gravitational field should also possess entropy. Recently, the idea has been proposed that gravitons can be separated from matter, and the number of gravitons is proportional to the square of the mass $N_{g}≃{\left(m/M_{p}\right)}^{2}$, providing an indirect constraint on gravitational entropy [31].

In the realm of gravitational entropy, various approaches have been formulated to define this elusive concept. Notably, gravitational entropy has been constructed from the Weyl curvature tensor, employing expressions such as $S=C^{abcd}C_{abcd}$ [32,33,34] or $S=C^{abcd}C_{abcd}/R^{ab}R_{ab}$ [35], where $C^{abcd}$ and $R^{ab}$ denote the Weyl and Ricci tensors, respectively. More recently, the Bel–Robinson tensor has been advocated as a foundation for defining gravitational entropy [36], offering a new perspective in this ongoing research. Furthermore, inspired by the profound area law governing black hole entropy, Grøn [30] derived an expression suggesting that the entropy density of the gravitational field scales with the magnitude of the gradient of a potential function, i.e., $s∼\vec{\nabla }\cdot \vec{\Psi }$. Building on these foundations and thermodynamic principles, we rigorously calculate the entropy density for a uniform gravitational field.

The paper is organized as follows: In Section 2, by constructing a uniform gravitational field model and leveraging Verlinde’s hypothesis, our calculations show that the entropy of the gravitational field is negative and proportional to the gravitational field strength. In Section 3, we calculate the entropy of the gravitational field in the outer space of a spherical object and extend the result to a Schwarzchild black hole, and we discuss the problem of black hole information loss. Finally the paper ends with some conclusions and discussions.

## 2. Entropy of the Gravitational Field

To calculate the entropy of the gravitational field, we first construct a uniform gravitational field. Consider an infinite plate with mass per unit area *m*; the gravitational field strength (acceleration) on both sides of the plate can be obtained as

(1) $$g=-2\pi Gm\int_{0}^{\infty }\frac{rx}{{\left(r^{2}+x^{2}\right)}^{3/2}}dr=-2\pi Gm.$$

Here, G is Newton’s gravitational constant. The gravitational field strength *g* is proportional to the plate’s surface mass density. The negative sign in the formula indicates that the gravitational field direction is towards the plate.

**Figure 1:**
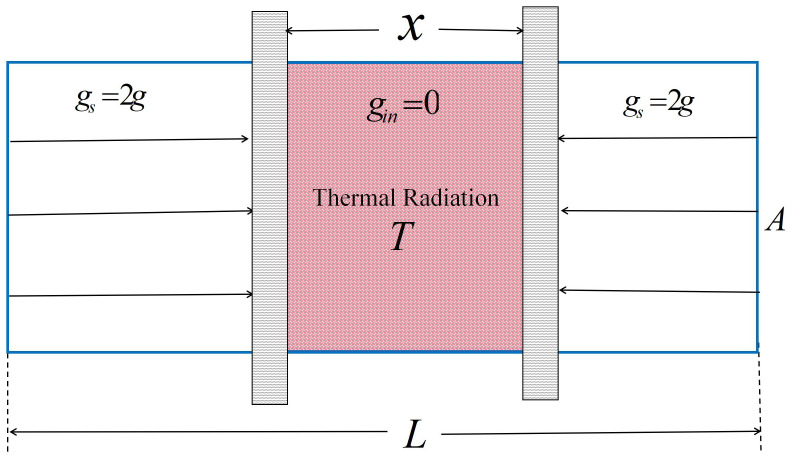
A cross-section view of our model. The two gray areas represent infinite parallel plates with mass surface density *m*; the gravitational field strength is $g_{\mathrm{in}}=0$ between the plates and $g_{s}$ outside them. A cylinder with cross-sectional area *A* perpendicular to the plate is selected as the research object.

For two identical infinite parallel plates, as shown in Figure 1, it is easy to show that the gravitational field is uniform $g_{s}=2g=-4\pi Gm$ on both sides of the parallel plates, while the internal field is zero $g_{\mathrm{in}}=0$. The volume occupied by the non-zero field increases as the plate separation decreases.

Suppose the region between the plates is filled with thermal radiation at temperature *T*. For the sake of analysis, we consider a cylinder of cross-sectional area *A* perpendicular to the plates. The cross-section view of the model is shown in Figure 1.

Since the energy of thermal radiation in the cylinder depends on the temperature *T* and the volume *V* of the thermal radiation, that is $U=aT^{4}V$, where $a=\pi^{2}k^{4}/15c^{3}\hbar^{3}$, the differential of *U* can be written as

(2) $$\begin{array}{cc} dU & ={\left(\frac{\partial U}{\partial T}\right)}_{x}dT+{\left(\frac{\partial U}{\partial x}\right)}_{T}dx,\\ \; & =4aAT^{3}xdT+aAT^{4}dx. \end{array}$$

where *x* represents the distance between the two parallel plates, *A* is the cross-sectional area of the cylinder, and k and ħ denote the Boltzmann constant and the reduced Planck constant, respectively.

As the plates approach each other, gravity performs positive work (dW=mgAdx). According to the law of energy conservation, all the positive work is converted into the energy of the thermal radiation in a quasi-static process, dU=dW=mgAdx. Combining this with Equation (2), one can obtain

(3) $$mgdx=4aT^{3}xdT+aT^{4}dx.$$

From Equation (3), we obtain

(4) $$dT=\frac{mg-aT^{4}}{4aT^{3}x}dx.$$

Since the entropy of thermal radiation is $S_{r}=4aT^{3}V/3$, the change in entropy of thermal radiation between two parallel plates can be obtained as

(5) $$\begin{array}{cc} dS_{r} & ={\left(\frac{\partial S_{r}}{\partial T}\right)}_{x}dT+{\left(\frac{\partial S_{r}}{\partial x}\right)}_{T}dx,\\ \; & =\left(\frac{aT^{3}}{3}+\frac{gm}{T}\right)Adx. \end{array}$$

Assuming a quasi-static process, the radiation pressure $P_{r}=aT^{4}/3$ must balance the gravitational force per unit area. Thus, $aT^{4}/3=-mg$. Substituting this into the expression for $dS_{r}$ gives $dS_{r}=0$, confirming that thermal radiation entropy remains constant during this quasi-static process.

Verlinde postulated that when a test particle with mass *m* moves by Δx towards a holographic screen, the entropy on the holographic screen increases by ΔS=2πkcmΔx/ħ [37]. We consider a holographic screen parallel to and located between the plates. When the plate separation decreases by dx, the entropy increase on this holographic screen is

(6) $$dS_{\mathrm{sc}}=\frac{2\pi kc}{\hbar }mAdx.$$

If the entropy of the whole system is equal to the entropy of the thermal radiation plus the entropy of the holographic screen, it would increase during the process ($dS_{r}+dS_{\mathrm{sc}}>0$). However, since the whole system is in an adiabatic quasi-static process, the total entropy of the system should remain constant; this violates the second law of thermodynamics. Since the only significant change is in the volume occupied by the gravitational field, we conclude that the gravitational field itself must possess entropy $S_{g}$ that changes. To satisfy the second law $dS_{r}+dS_{\mathrm{sc}}+dS_{g}=0$, the change in gravitational entropy density $s_{g}$ associated with field strength $g_{s}$ must be

(7) $$s_{g}=-\frac{dS_{\mathrm{sc}}}{Adx}=-\frac{kc}{\hbar G}\frac{\left|g_{s}\right|}{2}.$$

Here, $m=-g_{s}/4\pi G$ was utilized. This is the key result of the paper. Equation (7) represents the change in the entropy density of the gravitational field when the gravitational field changes, and the absolute value of the entropy density of a gravitational field cannot be determined by this method. It is obvious that the entropy density is proportional to the **magnitude of** gravitational acceleration $g_{s}$. Since entropy is a scalar quantity, an absolute value sign has been added to the gravitational acceleration $g_{s}$ in Equation (7). **Since acceleration is observer-dependent and can be transformed away by switching to a freely falling reference frame, Equation(7) implies that the entropy density vanishes. This is consistent with the third Law of thermodynamics, as the Unruh effect predicts a zero-field temperature in the absence of acceleration. Consequently, gravitational entropy is similarly observer-dependent.**

Interestingly, the entropy of the gravitational field is negative, and we all know that information is negative entropy, which means that the gravitational field carries information; this implies that the gravitational field carries information proportional to its strength. The entropy of a system can be obtained by integrating the entropy density over the volume,

(8) $$\begin{array}{cc} S_{g} & =\int s_{g}dV,\\ \; & =-\frac{kc}{2\hbar G}\int \left|g_{s}\right|dV. \end{array}$$

Suppose there exists a vector $\vec{\Psi }$ that satisfies $\left|g_{s}\right|=\nabla \cdot \vec{\Psi }$; then according to the Gaussian divergence theorem, it can be obtained as follows:

(9) $$\begin{array}{cc} S_{g} & =-\frac{kc}{2\hbar G}\int \nabla \cdot \vec{\Psi }dV,\\ \; & =-\frac{kc}{2\hbar G}\int_{\sigma }\vec{\Psi }\cdot \vec{d\sigma }. \end{array}$$

Since gravitational acceleration is equal to the negative gradient of the gravitational potential, the vector $\vec{\Psi }$ is closely related to the potential of the gravitational field. In particular, for the one-dimensional case, the magnitude of vector $\vec{\Psi }$ is equal to the magnitude of the gravitational potential.

Based on the area law of black hole entropy, Rudjord and his collaborators [38] proposed a gravitational entropy estimation considering the Bekenstant–Hawking entropy; they suggested that it can be expressed as

(10) $$S=k_{s}\int_{\sigma }\vec{\Psi }\cdot \vec{d\sigma },$$

where $\vec{\sigma }$ is the surface area of the event horizon of the black hole, and the vector function is defined as $\vec{\Psi }=P\vec{e_{r}}$, with $\vec{e_{r}}$ as a radial unit vector. The scalar function *P* is define using the Weyl and Kretschmann scalars, where

(11) $$P^{2}=\frac{C^{abcd}C_{abcd}}{R^{abcd}R_{abcd}}.$$

It was shown that while the Weyl–Kretschmann estimator reproduces the Hawking–Bekenstein entropy for a Schwarzschild black hole, it does not in the charged case [38,39]; a new definition of the scalar *P* could help to solve the problem. Our work provides a new clue for defining *P* from the perspective of thermodynamics. Henceforth, we adopt Planck units, where G=c=ħ=k=1

## 3. The Black Hole Entropy and the Black Hole Information Loss Problem

Consider a spherical mass *M*. When a test particle of mass *m* approaches it, Verlinde’s hypothesis states that the entropy on the holographic screen increases by

(12) $$dS_{\mathrm{sc}}=2\pi mdx.$$

Building upon this hypothesis and the holographic principle, Verlinde derived Newton’s law of gravitation and proposed that gravity is an entropic force [37]. We now analyze the evolution of the gravitational field and its entropy as *m* approaches *M*.

**Figure 2:**
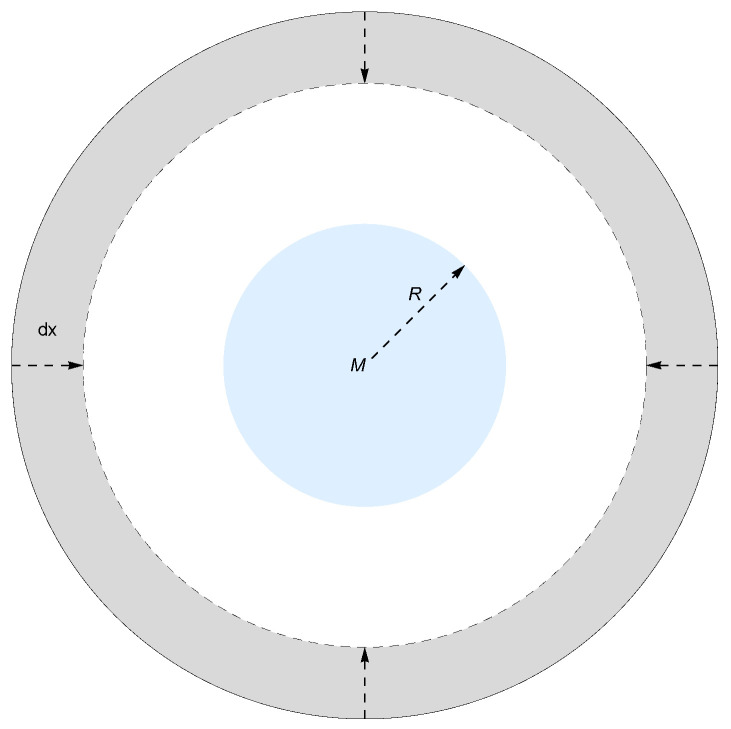
A spherical shell of mass *m* near a holographic screen enclosing an object of mass *M*. The gray region represents the volume dV swept by the shell’s displacement dx.

For analytical simplicity, we model the mass *m* as a thin, concentric spherical shell surrounding the central object *M*, as illustrated in Figure 2.

When the spherical shell *m* contracts its radius and approaches object *M* by a infinitesimal distance dx, the increase in entropy on an enclosing holographic screen is still given by Equation (12). The gravitational field strength changes only within the swept volume dV (i.e., the gray region in Figure 2), where $\Delta g=m/r^{2}$, while the gravitational field in the remaining space remains unaltered.

Assuming this process is adiabatic and quasi-static, the total entropy of the system must remain constant. The contraction of the shell increases the holographic screen entropy by $dS_{\mathrm{sc}}$, but since the gravitational field only varies within dV, the entropy associated with the field in this region must decrease to preserve the total entropy. Then, according to Equation (12), the change in gravitational entropy density in dV is

(13) $$\begin{array}{cc} \Delta s_{g} & =-\frac{dS_{\mathrm{sc}}}{4\pi r^{2}dx}\;,\\ \; & =-\frac{\Delta g}{2}. \end{array}$$

This result aligns with the earlier conclusion in Equation (7). The decrease in entropy accompanying the increase in field strength implies that the gravitational field carries negative entropy—effectively encoding information. Thus, the information content density of the gravitational field is proportional to its field strength.

The entropy of the gravitational field can be obtained by integrating over the space with respect to the entropy density of the gravitational field; then the total entropy of the gravitational field generated by a mass *M* can be expressed as

(14) $$S_{g}\left(M\right)=\int_{R}^{\infty }s_{g}4\pi r^{2}dr=-2\pi M\int_{R}^{\infty }dr.$$

where $s_{g}=-M/2r^{2}$. It is evident that this result is divergent. This divergence arises because gravity is a long-range force and space is infinite, causing the gravitational field entropy of even an infinitesimal mass δm object to diverge. To resolve this, we define the reference state such that when matter is uniformly distributed throughout infinite space, the gravitational entropy is maximized and set to zero. As matter localizes, the entropy of the gravitational field decreases. For a localized mass *M*, the entropy change relative to the reference state is

(15) $$\Delta S_{g}\left(M\right)=S_{g}\left(M\right)-\lim_{\delta m\to 0}\frac{M}{\delta m}S_{g}\left(\delta m\right)=-2\pi MR.$$

This conclusion is consistent with Bekenstein’s entropy constraint [40].

In particular, for a black hole of mass *M* with Schwarzschild radius R=2M, the entropy of the gravitational field outside the black hole is

(16) $$S_{\mathrm{BHg}}\left(M\right)=-4\pi M^{2}.$$

The corresponding information stored in the gravitational field is $I_{\mathrm{BHg}}=4\pi M^{2}$, which is equal to the entropy of the Schwarzschild black hole itself, where $S_{\mathrm{BHg}}\left(M\right)=4\pi M^{2}$.

(17) $$S_{\mathrm{BHg}}\left(M\right)+S_{\mathrm{BH}}\left(M\right)=0.$$

This cancellation suggests a profound connection between black hole entropy and the gravitational field. When an object falls into a black hole, the entropy of the external gravitational field decreases (i.e., its information content increases). Since $I_{\mathrm{BHg}}=S_{\mathrm{BH}}$, all information associated with the black hole is stored in its external gravitational field [41].

During black hole evaporation, when a particle of mass *m* in the black hole is radiated away via Hawking emission, the external gravitational field weakens, reducing its information content by [41,42]

(18) ΔI(M,m)=8π(M−m/2)m.

This transfer of information from the gravitational field to the radiation is mediated by gravitational correlations. According to the tunneling method [16], the tunneling probability rate is given by the following expression:

(19) Γ(M,m)=exp[−8π(M−m/2)m].

Obviously, this probability rate deviates from a purely thermal behavior, contrasting with the simpler thermal case where Γ∼exp(−8πMm). Specifically, the deviation indicates that the radiated particles carry information from the black hole’s gravitational field via gravitational interactions. When the black hole evaporates completely, the total information recovered matches the initial entropy $I\left(M\right)=4\pi M^{2}$, thereby ensuring the conservation of information in black hole dynamics [17,18,19].

## 4. Entropic-Accelerating Universe

The concept of an accelerating universe driven by entropy has emerged as a compelling alternative to traditional dark energy models. Several theories, such as entropic cosmology and entropic-force models, propose that the acceleration of cosmic expansion is due to the increase in entropy at the cosmic horizon, leading to an “entropy force” that drives the expansion. These models aim to explain the observed acceleration without invoking dark energy or the cosmological constant, offering a new thermodynamic perspective on cosmic dynamics. For instance, Easson, Frampton, and Smoot suggest that the entropy associated with the cosmic horizon generates a force that accelerates the universe’s expansion, and they present a phenomenological model based on surface terms that fits supernova data well [43]. Similarly, Zamora and Tsallis develop a thermodynamically consistent entropic model that explains late-time cosmic acceleration without dark energy, showing good agreement with supernova data and Hubble parameter measurements [44].

We now calculate the gravitational field entropy of the universe and investigate its potential role in cosmic acceleration. According to Hubble’s law, the recession velocity of a galaxy is given by

(20) v=Hr,

where $H=\dot{a}/a$ denotes the Hubble parameter (*a* is the scale factor of the universe), while *r* is the proper distance between the galaxy and the observer. The cosmic acceleration g(r) experienced by the galaxy can be derived by differentiating the velocity with respect to time, with

(21) $$g\left(r\right)=\frac{dv}{dt}=\left(H^{2}+\dot{H}\right)r,$$

where $\dot{H}$ is the time derivative of the Hubble parameter.

Using our gravitational entropy density result from Equation (7), the total entropy of the cosmic gravitational field within the Hubble volume (up to the Hubble radius, where $R_{H}=c/H$) is calculated as

(22) $$\begin{array}{cc} S_{U} & =\int_{0}^{R_{H}}g\left(r\right)dV,\\ \; & =\pi R_{H}^{2}+\pi \dot{H}R_{H}^{4}. \end{array}$$

This expression for $S_{U}$, which explicitly depends on $R_{H}$ and $\dot{H}$, differs from previous formulations in the literature [43,45]. As the Hubble radius $R_{H}$ evolves, the cosmic entropy $S_{U}$ changes accordingly. The associated entropic force $F_{s}$ is given by

(23) $$\begin{array}{cc} F_{s} & =-T\frac{dS_{U}}{dR_{H}},\\ \; & =-H\left(R_{H}+2\dot{H}R_{H}^{2}\right). \end{array}$$

where we have adopted the horizon temperature T=H/2π. The pressure associated with this entropic force is

(24) $$P_{s}=\frac{F_{s}}{4\pi R_{H}^{2}}=-\frac{H^{2}+2\dot{H}}{4\pi }.$$

To incorporate this entropic pressure $P_{s}$ into cosmology, we modify the Friedmann equation by defining an effective pressure $P_{\mathrm{eff}}=P+P_{s}$, where *P* encompasses ordinary matter, dark matter, and radiation pressures. Substituting $P_{\mathrm{eff}}$ into the standard Friedmann equation yields the following:

(25) $$\frac{\ddot{a}}{a}=-\frac{4\pi }{3}\left(\rho +3P_{\mathrm{eff}}\right),$$

where ρ is the total energy density of the universe. By substituting $P_{\mathrm{eff}}$ into the equation, we obtain

(26) $$\frac{\ddot{a}}{a}=-\frac{4\pi }{3}\left(\rho +3P\right)+\left(H^{2}+2\dot{H}\right).$$

Recall that the standard relation between the scale factor and the Hubble parameter gives $\ddot{a}/a=H^{2}+\dot{H}$, Substituting this into Equation (26) simplifies it to

(27) $$\dot{H}=\frac{4\pi }{3}\left(\rho +3P\right).$$

For ordinary matter, dark matter, and radiation, the energy–momentum condition ρ+3P>0 always holds. Combining Equations (27) and (26) shows that the universe undergoes accelerated expansion even without dark energy, driven primarily by the entropic force. As the universe expands, the energy density ρ and pressure *P* decrease monotonically, causing $\dot{H}$ to approach zero. In the late universe, the Hubble parameter thus tends to a constant, leading to exponential expansion of the scale factor $a\left(t\right)=a_{0}e^{H(t-t_{0})}$.

## 5. Conclusions and Discussion

In this study, we constructed an ideal model of a uniform gravitational field and, based on Verlinde’s entropy increment hypothesis, rigorously demonstrated that the entropy density of the gravitational field is proportional to the gravitational acceleration. A key finding is the negative gravitational entropy, implying an information storage capacity within the field proportional to its strength. Applying this result to Schwarzschild black holes, we showed that the entropy of a black hole is equivalent to the information stored in the gravitational field outside its event horizon. This indicates that the information traditionally thought to be “confined within the black hole” is actually encoded in its external gravitational field. During black hole evaporation, radiated particles transmit this information through gravitational correlations, resulting in a non-thermal spectrum of Hawking radiation—consistent with the tunneling model predictions [16,17].

Although our derivation of gravitational field entropy was performed under quasi-static thermal equilibrium conditions, since entropy is a state function (independent of the specific process), this conclusion holds universally, regardless of whether the system undergoes a quasi-static process. Our framework naturally yields T=g/2π for the gravitational field temperature, aligning with the Unruh effect prediction. This consistency further validates the robustness of our framework.

The second law requires a low-entropy initial state for the universe. This appears paradoxical, as the standard Big Bang model describes the early universe as nearly homogeneous, isotropic, and uniformly distributed in temperature—properties typically associated with high entropy [30]. However, this contradiction is resolved by accounting for gravitational entropy: in the early universe, where matter was densely concentrated, the gravitational field contributed a large negative entropy; when integrated into the total entropy budget, this negative gravitational entropy places the early universe a low-entropy state, satisfying the second law.

Notably, we observe a striking distinction in entropy scaling: the entropy of a uniform gravitational field is proportional to its volume, whereas the entropy of gravitational fields generated by horizon-bearing sources (e.g., black holes) scales with the surface area—consistent with the black hole entropy area law. Extended to cosmology, our gravitational entropy expression, combined with the entropy increase principle, explains the observed cosmic acceleration without dark energy. This suggests that entropic forces may play a fundamental role in cosmic dynamics [45,46,47].

While our work provides a thermodynamic framework for understanding gravitational entropy and black hole information storage, several questions remain. For instance, the generalization of our results to rotating (Kerr) or charged (Reissner-Nordström) black holes requires further investigation, as their gravitational field structures and entropy properties may introduce new complexities. Additionally, the microscopic origin of gravitational field entropy—such as its connection to gravitons or holographic degrees of freedom—warrants deeper exploration to solidify the link between thermodynamics and quantum gravity. Nevertheless, our findings offer a new perspective on resolving the black hole information loss paradox and shed light on the role of gravitational entropy in cosmic evolution, bridging key gaps between quantum theory, gravity, and thermodynamics.

## Data Availability

The original contributions presented in this study are included in the article. Further inquiries can be directed to the corresponding author.
